# Core outcome set to assess effectiveness in multimodal pain therapy – preliminary results of an interdisciplinary online survey

**DOI:** 10.1186/1745-6215-16-S1-P1

**Published:** 2015-05-29

**Authors:** Christian Kopkow, Stefanie Deckert, Jochen Schmitt, Rainer Sabatowski, Ulrike Kaiser

**Affiliations:** 1 Center for Evidence-based Healthcare, Dresden, Germany; 2 Comprehensive Pain Center, Dresden, Germany

## Background

For multimodal pain therapy (MPT), a bio-psycho-social therapy approach for patients suffering from chronic pain, a core outcome set (COS) is currently lacking. Following the recommendations from initiatives such as Outcome Measures in Rheumatology (OMERACT) and Harmonizing Outcome Measures for Eczema (HOME), the study “Validation and Application of a patient relevant core set of outcome domains to assess multimodal PAIN therapy” (VAPAIN) aims to develop an accepted and valid COS for MPT.

## Methods

An online survey consisting of two rounds was conducted using the mOMEnt software provided by the COMET initiative. 25 stakeholders in MPT (patient representatives, physicians, physiotherapists, psychotherapists and methodological experts) were nominated by their associations and asked to participate. During both rounds, participants were asked to rate the importance of proposed domains regarding a) effectiveness trials (ES) and b) daily record keeping (DRK). Domains to be rated were identified through a systematic review of reported outcomes in MPT studies. During round 1, survey participants were allowed to name additionally relevant outcomes and were asked to name the number of minimal/maximal number of outcome domains to be included in the COS. During round 2 participants received feedback regarding their own previous ratings and also regarding ratings from other participants had to name which outcomes should be included in the COS.

## Results

All participants completed round 1, but only 22 round 2 (88% response rate). The maximal and minimal number of outcome domains to be included in the COS ES was 9 and 4, and for the COS DRK 6 and 3. Pain intensity/severity (18/22), health-related quality of life (17/22), and pain-related disability (11/22) was named most frequently by the survey participants as being part of the COS for ES. Pain intensity (15/22), psychological distress (9/22) and both analgesic medication intake and pain-related disability (7/22) were named for the COS DRK.

## Conclusions

The results of this online survey regarding outcomes in studies evaluating MPT are mostly in line with the results from the preceding systematic review on outcome domains. Furthermore, the domains for the COS for effectiveness trials and daily record keeping were not fundamentally different. The identified preliminary outcome domains will be consented during a face-to-face meeting (consensus conference) in a moderated group discussion to determine a COS for MPT.

